# Auxin-inducible protein depletion system in fission yeast

**DOI:** 10.1186/1471-2121-12-8

**Published:** 2011-02-11

**Authors:** Mai Kanke, Kohei Nishimura, Masato Kanemaki, Tatsuo Kakimoto, Tatsuro S Takahashi, Takuro Nakagawa, Hisao Masukata

**Affiliations:** 1 Graduate School of Science, Osaka University, 1-1 Machikaneyama-cho, Toyonaka, Osaka 560-0043, Japan; 2 Current Address: National Institute of Genetics, Yata 1111, Mishima, Shizuoka 411-8540, Japan

## Abstract

**Background:**

Inducible inactivation of a protein is a powerful approach for analysis of its function within cells. Fission yeast is a useful model for studying the fundamental mechanisms such as chromosome maintenance and cell cycle. However, previously published strategies for protein-depletion are successful only for some proteins in some specific conditions and still do not achieve efficient depletion to cause acute phenotypes such as immediate cell cycle arrest. The aim of this work was to construct a useful and powerful protein-depletion system in *Shizosaccaromyces pombe*.

**Results:**

We constructed an auxin-inducible degron (AID) system, which utilizes auxin-dependent poly-ubiquitination of Aux/IAA proteins by SCF^TIR1 ^in plants, in fission yeast. Although expression of a plant F-box protein, TIR1, decreased Mcm4-aid, a component of the MCM complex essential for DNA replication tagged with Aux/IAA peptide, depletion did not result in an evident growth defect. We successfully improved degradation efficiency of Mcm4-aid by fusion of TIR1 with fission yeast Skp1, a conserved F-box-interacting component of SCF (improved-AID system; *i*-AID), and the cells showed severe defect in growth. The *i*-AID system induced degradation of Mcm4-aid in the chromatin-bound MCM complex as well as those in soluble fractions. The *i*-AID system in conjunction with transcription repression (*off*-AID system), we achieved more efficient depletion of other proteins including Pol1 and Cdc45, causing early S phase arrest.

**Conclusion:**

Improvement of the AID system allowed us to construct conditional null mutants of *S. pombe*. We propose that the *off*-AID system is the powerful method for *in vivo *protein-depletion in fission yeast.

## Background

*Schizosaccharomyces pombe *is a widely used model organism for analysis of important cellular functions [1-3]. The use of conditional inactivation by mutations or depletion of proteins *in vivo *has been used successfully for analysis of gene functions. A conditional protein degradation system, so-called "degron", which depletes proteins from cells, is a powerful tool for analyzing the "null" phenotype of various genes. In budding yeast, a heat-inducible degron (*ts*-degron) system has been devised [4,5] and used for studies of essential gene functions [6,7]. In fission yeast, the *ts-*degron mutant of Bir1, a nuclear protein involved in mitotic segregation, has been reported to cause destruction of the protein, resulting in growth defects at restrictive temperature [8]. For functional analysis, however, as this system is unable to deplete proteins sufficiently to arrest the cell cycle, it is often combined with *ts-*alleles of the genes of interest [9,10].

Recently, an auxin-inducible degron (AID) system was developed for use in budding yeast and higher eukaryotic cells [11]. This strategy involves a plant-specific mechanism that relies on response to the plant hormone auxin and a conserved poly-ubiquitination pathway involving the E3 ubiquitin ligase, SCF (Skp1, Cullin and F-box protein) complex [12,13]. In plant cells, auxin binds to transport inhibitor response 1 (TIR1) protein [14,15] and promotes binding of the SCF^TIR1^, a form of SCF containing TIR1, to Aux/IAA transcription repressors [16-18]. The Aux/IAA proteins are poly-ubiquitinated by the SCF^TIR1 ^and then degraded by proteasomes [19]. Except for the auxin-dependent recognition of Aux/IAA proteins by TIR1, components of the SCF and ubiquitin-proteasome pathway, especially F-box interacting protein skp1, are conserved among eukaryotes. This makes it possible to form the SCF^TIR1 ^in non-plant cells by expression of TIR1 and to degrade Aux/IAA-tagged proteins depending on addition of auxin.

We have constructed an AID system for use in *S. pombe *by expressing TIR1. Although Mcm4 protein fused with Aux/IAA peptide (aid-tag) at the C-terminus (Mcm4-aid) was decreased upon addition of auxin, the strain did not show any obvious growth defect because of inefficient depletion. Here we describe an improved form of the AID system. Depletion of Mcm4-aid protein was greatly enhanced by expression of Skp1-TIR1, a fusion protein comprising plant TIR1 and fission yeast Skp1. This *i*-AID system (improved AID system) was applicable for depletion of other essential replication proteins including Orc2, Cdc45 and Pol1. Furthermore, in conjunction of the *i*-AID system with transcription repression (*off*-AID system), Cdc45- or Pol1-depleted cells showed severe replication defects resulting in cell cycle arrest. The *off*-AID system involving two-step gene modifications will be a powerful tool for analyzing the function of essential genes under null conditions in *S. pombe*.

## Results

### Auxin-dependent degradation of Mcm4-aid in fission yeast

**Figure 1:**
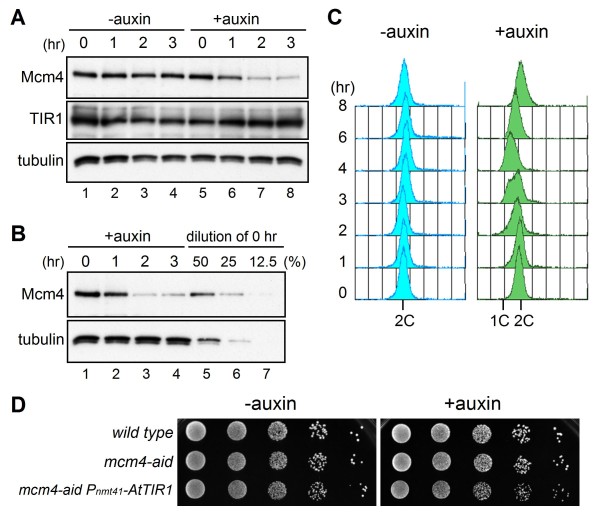
**Auxin-induced degradation of Mcm4-aid**. (A) Amounts of Mcm4-aid and TIR1 in the presence or absence of auxin. HM1905 *mcm4-aid P*_*nmt41*_*-AtTIR1 *cells were cultured at 25°C in the presence (lanes 5-8) or absence (lanes 1-4) of auxin (0.5 mM) for indicated periods and proteins in whole-cell extracts were separated in 7.5% polyacrylamide gel and analyzed by immunoblotting with anti-Mcm4, myc (TIR1), and TAT1 (tubulin) antibodies. (B) The amount of Mcm4-aid after depletion was compared to that in the 0 h sample diluted with SDS sample buffer to 50%, 25%, and 12.5%. (C) Flow cytometry analysis of *mcm4-aid P*_*nmt41*_*-AtTIR1 *cells. HM1905 *mcm4-aid P*_*nmt41*_*-AtTIR1 *cells were cultured at 25°C and collected every 1 h after addition of auxin (0.5 mM), and then the DNA contents of the cells were analyzed. Positions of 1C and 2C DNA contents are indicated. (D) Growth on auxin plates. Log-phase cultures of wild type, *mcm4-aid *without TIR1 (HM1909) and *mcm4-aid P*_*nmt41*_*-AtTIR1 *(HM1905) were serially diluted and spotted onto EMM plates with or without auxin (0.5 mM) and incubated at 25°C.

To develop an auxin-inducible protein degradation system in fission yeast, we constructed a strain that expresses *Arabidopsis thaliana *TIR1 (AtTIR1), a plant specific F-box protein that binds auxin, from the chromosome. We placed the *AtTIR1 *gene under control of the *nmt41 *(*P*_*nmt41*_) promoter to induce gene expression in the absence of thiamine [20,21]. As a target of degradation, we used Mcm4, a component of the MCM complex, which is required throughout the whole process of chromosomal DNA replication. The *mcm4-aid*, tagged with full-length IAA17 (229-amino acid peptides) at the C-terminus of the *mcm4*^+ ^gene on the chromosome, grew as well as the wild-type strain without the aid-tag (data not shown). The level of Mcm4-aid protein analyzed by immunoblotting with anti-Mcm4 antibody was similar to that in the untagged strain (data not shown). Upon addition of a synthetic auxin, NAA (1-naphthaleneacetic acid), to log-phase cells expressing AtTIR1, the protein level of Mcm4-aid decreased in 1 h (Figure 1A). The amount of protein at 2 h was estimated to be about 25% of that without auxin (Figure 1B). To examine whether reduction of Mcm4-aid protein affects DNA replication and cell growth, the DNA content was analyzed by flow cytometry (Figure 1C). In the absence of auxin, *mcm4-aid *cells retained a 2C DNA content, because fission yeast cells have a long G2 phase and cytokinesis occurs in S phase (Figure 1C, left panel). In contrast, upon addition of auxin, cells with less than a 2C DNA content appeared at 2-4 h (Figure 1C, right panel), suggesting that a decrease in Mcm4-aid protein retarded DNA replication. However, these cells did not exhibit a severe growth defect on the auxin-containing medium (Figure 1D). These results show that, although the AID system degrades Mcm4-aid protein in fission yeast, more efficient depletion of the target protein is required for physiological analysis.

### NLS and Skp1-fusion to AtTIR1 enhances the efficiency of the AID system

**Figure 2:**
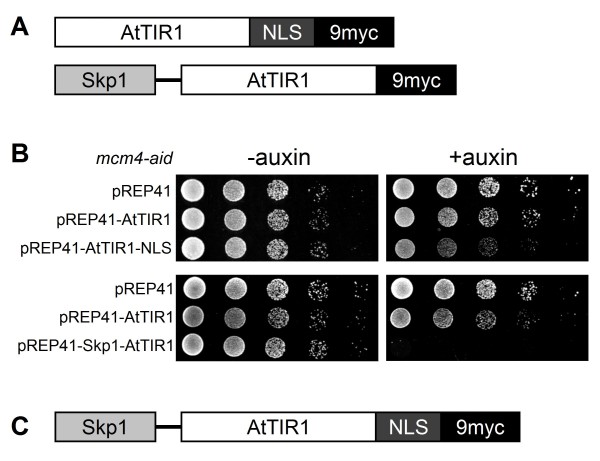
**Fusion of Skp1 and NLS to TIR1 enhances growth defects of *mcm4-aid *in the presence of auxin**. (A) Schematic illustrations of AtTIR1 genes fused with NLS and Skp1. Two copies of NLS sequences derived from SV40 were inserted between AtTIR1 and 9myc tag (upper), while fission yeast Skp1 (SpSkp1) with 16-amino-acid linker peptides (see Methods) was fused to AtTIR1 at the N-terminus (lower). (B) Serially diluted cultures of HM1910 *mcm4-aid leu1-32 *cells carrying the pREP41D vector alone (MKF53), pREP41D-AtTIR1 (MKF54), pREP41D-AtTIR1-NLS (MKF55) and pREP41D-Skp1-AtTIR1 (MKF60) were spotted onto EMM plates containing auxin (0.5 mM) and incubated at 25°C. (C) Schematic illustration of the Skp1-AtTIR1- NLS-9myc fusion is presented.

We considered two possible reasons for the insufficient depletion of Mcm4-aid protein. First, the concentration of AtTIR1 imported to the nucleus may not be sufficient to promote efficient degradation, since the MCM complex exists throughout the cell cycle. If this is the case, addition of nuclear localization signals (NLSs) to AtTIR1 should increase the efficiency of degradation. Second, the plant AtTIR1 may fail to interact strongly with the *S. pombe *SCF components. This problem could be overcome by fusion of AtTIR1 with fission yeast Skp1, a conserved F-box-interacting protein [22] (Figure 2A). The pREP41 plasmid carrying AtTIR1-NLS or Skp1-AtTIR1 was introduced into the *mcm4-aid *strain, and the growth of the cells was examined on plates containing auxin. Cells expressing AtTIR1-NLS grew more slowly than those expressing AtTIR1 in the presence of auxin (Figure 2B). Cells harboring Skp1-AtTIR1 exhibited severe growth defects on the auxin plate (Figure 2B). These results suggest that the inefficient degradation of Mcm4-aid protein was mainly due to inefficient formation of the SCF^TIR1 ^complex in fission yeast. Therefore, we used a strain carrying a single copy of *skp1-AtTIR1-NLS *fusion gene (Figure 2C) inserted into the genome in the following experiments.

### Optimizing the expression of Skp1-AtTIR1-NLS from the genomic locus

**Figure 3:**
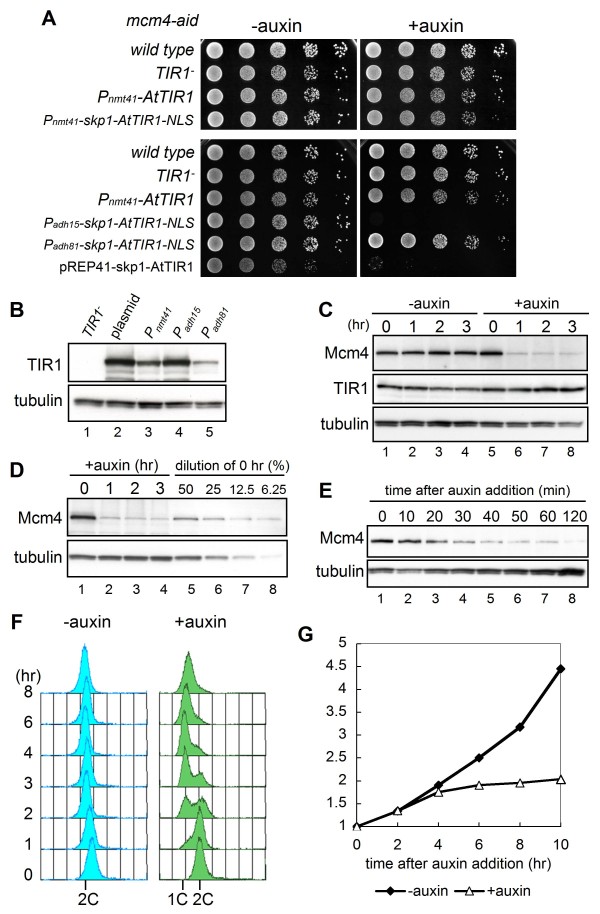
**The improved-AID system (*i*-AID system) promotes efficient Mcm4-aid degradation resulting in acute defect in DNA replication and cell cycle arrest**. (A) *mcm4-aid *cells expressing AtTIR1 under control of the *P*_*nmt41 *_promoter and Skp1-AtTIR1-NLS under control of the *P*_*nmt41*_, *P*_*adh15 *_or *P*_*adh81 *_promoter located on the chromosome were spotted onto EMM plates containing auxin (0.5 mM) and incubated at 25°C. (B) *mcm4-aid *cells harboring the pREP41D-Skp1-AtTIR1 plasmid (MKF60) and those carrying *P*_*nmt41*_*-skp1-AtTIR1-NLS *(HM2491), *P*_*adh15*_*-skp1-AtTIR1-NLS *(HM2473) and *P*_*adh81*_*-skp1-AtTIR1-NLS *(HM2475) on the chromosome were grown in EMM, and the amount of TIR1 in the extracts was analyzed by 7.5% polyacrylamide gel electrophoresis followed by immunoblotting with anti-myc (TIR1) and anti-TAT1 (tubulin) antibodies. (C) Whole-cell extracts of *mcm4-aid P*_*adh15*_*-skp1-AtTIR1-NLS *cells (HM2473) were prepared every 1 h after addition of auxin (0.5 mM) at 25°C. Proteins in whole-cell extracts were separated in 7.5% polyacrylamide gel and analyzed by immunoblotting with anti-Mcm4, anti-myc (TIR1), and anti-TAT1 (tubulin) antibodies. (D) The amount of Mcm4-aid protein after depletion was compared with that in the 0 h sample diluted to 50%, 25%, 12.5% and 6.25%. (E) Whole-cell extracts of *mcm4-aid P*_*adh15*_*-skp1-AtTIR1-NLS *cells (HM2473) were prepared at indicated time point after addition of auxin at 25°C and proteins were analyzed as in (C). (F) DNA contents of HM2473 *mcm4-aid P*_*adh15*_*-skp1-AtTIR1-NLS *cells in the absence (left) or presence (right) of auxin were analyzed by flow cytometry. (G) Relative numbers of HM2473 *mcm4-aid P*_*adh15*_*-skp1-AtTIR1-NLS *cells with or without auxin were measured at the indicated time points by Sysmex (Sysmex Co., Kobe, Japan) [46].

During the course of improving the AID system, we found that the expression level of TIR1 was crucial for efficient depletion of Mcm4-aid protein. The strain carrying *P*_*nmt41*_-*skp1-AtTIR1-NLS *integrated at the *ade6*^+ ^locus on the chromosome did not show marked growth defects on auxin plates, unlike the results obtained with the strain harboring *P*_*nmt41*_-*skp1-AtTIR1 *on a multicopy plasmid (Figure 3A). Immunoblotting revealed that the degree of Skp1-AtTIR1-NLS expression from the *P*_*nmt41 *_promoter on the chromosome was about half that of Skp1-AtTIR1 expressed on the plasmid (Figure 3B), suggesting that a certain level of TIR1 expression is required for efficient degradation. Comparison of TIR1 expression from different promoters showed that the amount of Skp1-AtTIR1-NLS expressed under control of the *adh15 *promoter (*P*_*adh15*_), a weak derivative of the *adh1 *promoter [23], was similar to that of Skp1-AtTIR1 expressed from the *P*_*nmt41 *_promoter on the plasmid, while the expression form *P*_*adh81*_, a much weaker derivative of *P*_*adh1 *_[24] was less than that from *P*_*nmt41 *_(Figure 3B). The *P*_*adh15*_*-skp1-AtTIR1-NLS mcm4-aid *strain showed markedly defective growth on auxin-containing plates, whereas the *P*_*adh81 *_derivative did not (Figure 3A). However, a further increase in TIR concentration, such as that achieved under control of the *nmt1 *or *adh1 *promoter, led to inhibition of cell growth even in the absence of the aid-tag and auxin (data not shown). We did not observe inhibition in specific cell-cycle phase. Therefore, the cellular concentration of TIR1 is important for efficient depletion of the target protein as has been observed in plant cells [14,25].

To examine how efficiently Mcm4-aid protein was degraded in the *P*_*adh15*_*-skp1-AtTIR1-NLS mcm4-aid *strain, the amount of Mcm4-aid in cell extracts prepared from log-phase cells in the presence or absence of auxin was analyzed by immunoblotting. The amount of Mcm4-aid protein decreased rapidly within 1 h to below 12.5% of the original amount (Figure 3C and 3D). The decrease was detected as early as 20 min after addition of auxin (Figure 3E). Under such conditions, a sharp 1C DNA peak appeared and was still evident at 8 h after addition of auxin (Figure 3F). The cell number did not increase after one cell division (Figure 3G) and cells were arrested with elongated shape containing a single nucleus (data not shown). These results show that the improved AID (*i*-AID) system depleted Mcm4-aid protein to cause tight cell cycle arrest at early S phase.

### Degradation of Mcm4-aid protein in the chromatin-bound MCM complex

**Figure 4:**
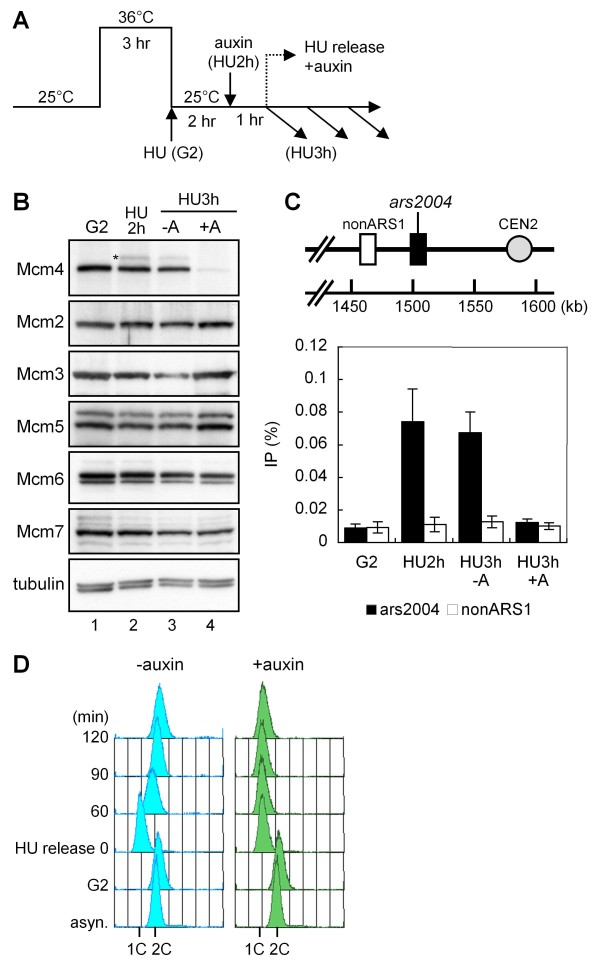
**Efficient depletion of Mcm4-aid protein in the MCM complex on chromatin causes dissociation of Mcm6 from chromatin and replication fork arrest**. (A) Experimental scheme. HM3086 *cdc25-22 mcm4-aid P*_*adh15*_*-skp1-AtTIR1-2NLS *cells were arrested at the G2/M boundary by incubation at 36°C (indicated as G2) and released at 25°C in the presence of hydroxyurea (HU) (12 mM) to arrest them at early S-phase. After 2 h of incubation with HU (HU2h), the cells were incubated with or without auxin (0.5 mM) for 1 h (HU3h+A, HU3h-A). After removal of HU by filtration, the cells were cultured with or without auxin (0.5 mM) and their DNA contents were analyzed. (B) Immunoblotting of MCM components. The amounts of proteins in the extracts prepared at G2, HU2h, and HU3h with or without auxin were analyzed by immunoblotting with antibodies against Mcm4, Mcm2, Mcm3, Mcm5, Mcm6, Mcm7 and TAT1. Position of phosphorylated Mcm4 in the presence of HU is indicated by an asterisk (*). (C) Dissociation of Mcm6 from the replication origin by depletion of Mcm4-aid. Positions of *ars2004 *and non-ARS1 are indicated above the panel with the distance from the left end of chromosome II. ChIP assay with anti-Mcm6 was carried out at G2, HU2h, HU3h-A (-auxin) and HU3h+A (+auxin). Recoveries of *ars2004 *and non-ARS1 fragments measured by real-time PCR are presented. The error bar shows the standard deviations for three independent ChIP analyses. (D) DNA contents of cells released from HU block upon depletion of Mcm4. Cells released from HU block were incubated in the presence or absence of auxin (0.5 mM), and the DNA contents were analyzed. Positions of 1C and 2C DNA contents are shown.

Next, we investigated whether the *i*-AID system promotes degradation of Mcm4-aid protein in the chromatin-bound MCM complex. Cells expressing Mcm4-aid and Skp1-AtTIR1-NLS were synchronized at the G2/M boundary by *cdc25-22 *mutation [26] and released in the presence of hydroxyurea, HU, which allows replication to initiate but retards fork progression due to depletion of deoxynucleotide pools (Figure 4A). The cells were then incubated for 1 h with auxin, and the amount of Mcm4-aid protein was analyzed by immunoblotting. The amount of Mcm4-aid was greatly decreased in cells treated with auxin (Figure 4B, HU3h+A, lane 4), relative to that in the absence of auxin (HU3h-A, lane 3). These results indicate that most of the Mcm4-aid protein was efficiently degraded in the HU-arrested cells. The band showing decreased mobility (as indicated by an asterisk in lane 2 of Figure 4B) at 2 h in the presence of HU was probably a form of Mcm4-aid protein phosphorylated by DDK, CDK or checkpoint kinases activated in the presence of HU [27-29]. We were concerned whether the *i*-AID system decreased the amounts of other subunits of the MCM complex in addition to Mcm4-aid protein. However, the amounts of these subunits did not change significantly in the presence of auxin (Figure 4B). It has been shown that the individual subunits of the MCM complex exists in similar amounts [30], and that the majority form heteromeric hexamers in fission yeast cells [31]. Taking these observations together with our present results, it is likely that Mcm4-aid protein is selectively degraded, leaving the other subunits undegraded.

If Mcm4-aid is degraded in the chromatin-associated MCM complex, the other subunits may not remain on the chromatin. To examine whether the other MCM subunits were released upon degradation of Mcm4-aid protein, we carried out a chromatin immnoprecipitation (ChIP) assay using anti-Mcm6 antibody. Two sets of primers amplifying the *ars2004 *locus, an efficient replication origin [32], and a non-ARS1 locus, 30 kb distant from the origin, were used for real-time PCR to measure the relative amounts of precipitated DNA (Figure 4C). In HU-arrested cells without auxin (HU2h, HU3h-A), Mcm6 was enriched at *ars2004 *in comparison with non-ARS1, while no significant localization was observed in G2-arrested cells (G2) (Figure 4C), as shown in a previous study [33]. Upon addition of auxin, IP recovery of *ars2004 *was decreased markedly to a level similar to that of non-ARS1 (Figure 4C, HU3h+A). These results indicate that Mcm4 is required for maintenance of the other MCM subunits on the chromatin. From these results, we concluded that the *i*-AID system induced efficient degradation of Mcm4-aid in the chromatin-bound MCM complex.

As the MCM complex is essential for progression of the replication fork, dissociation of the MCM complex will arrest DNA replication. To confirm this, we used flow cytometry to analyze the recovery of DNA replication upon removal of HU (Figure 4D). In the absence of auxin, DNA replication readily resumed after removal of HU, as DNA the content increased from 1C to 2C in 0-90 min (Figure 4D, left panel). In contrast, the DNA content of cells treated with auxin remained at 1C even at 120 min after removal of HU (Figure 4D, right panel), indicating that depletion of Mcm4 prevented progression of the replication fork. This is consistent with the above conclusion that Mcm4 in the chromatin-bound MCM complex is efficiently depleted by the *i*-AID system.

### Application of the *i*-AID system for depletion of other essential proteins in fission yeast

**Figure 5:**
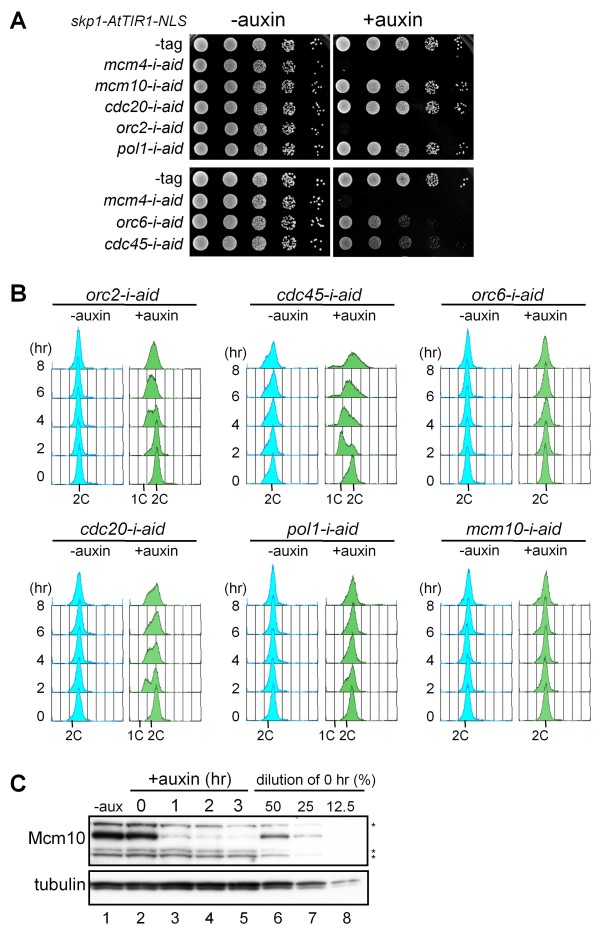
**Application of the *i*-AID system to other replication factors**. (A) Derivatives of the *P*_*adh15*_*-skp1-AtTIR1-NLS *strain carrying *mcm4-aid *(HM2473), *mcm10-aid *(HM2550), *cdc20-aid *(HM2551), *orc2-aid *(HM2572), *pol1-aid *(HM2552), *orc6-aid *(HM2575) and *cdc45-aid *(HM2578) were spotted on EMM plates containing auxin (0.5 mM) and incubated at 25°C. (B) Flow cytometry analysis of the derivatives shown in (A). Each strain was incubated in the presence of auxin (0.5 mM) at 25°C and the DNA contents were analyzed. (C) Immunoblotting analysis of Mcm10-aid. HM2550 *P*_*adh15*_*-skp1-AtTIR1-NLS mcm10-aid *cells incubated in the presence of auxin (0.5 mM) at 25°C and cell extracts prepared at the indicated time points were subjected to immunoblotting with anti-Mcm10 and anti-TAT1 antibodies. The 0 h sample was diluted to 50%, 25% and 12.5% with SDS sample buffer, and used for comparison of the protein amounts. Non-specific protein bands are indicated by asterisks (*).

In order to examine whether the *i*-AID system is applicable for depletion of other proteins in fission yeast, we constructed strains expressing various essential factors fused with the aid-tag at the C-terminus. The factors examined were Orc2, Orc4 and Orc6, components of the origin recognition complex (ORC), Cdc45, Mcm10, Psf1, a subunit of GINS, Cdc20 and Dpb2, the catalytic and the second largest subunits of DNA Polε, Pol1, Pol12 and Spp2, the catalytic, the second largest and the primase subunits of DNA Polα, Hsk1, the catalytic subunit of DDK, Ssl3, a subunit of cohesin loader, and Cia1/Asf1, a histone chaperone. The effects of the *i*-AID system were evaluated in terms of growth on auxin-containing plates and DNA content analyzed by flow cytometry. Growth of the *orc2-i-aid *strain was severely retarded on the auxin plate and *orc6-i-aid *and *cdc45-i-aid *strains showed slow growth. On the other hand, no significant growth retardation was observed for strains *cdc20-i-aid*, *pol1-i-aid *and *mcm10-i-aid *(Figure 5A and Table 1). The results of flow cytometry showed that DNA replication was retarded in *orc2-i-aid*, *cdc45-i-aid *and *cdc20-i-aid*, and slightly delayed in *orc6-i-aid *and *pol1-i-aid*, whereas no significant defect was observed for *mcm10-i-aid *(Figure 5B and Table 1). Strains *orc4-i-aid*, *psf1-i-aid*, *pol12-i-aid*, *spp2-i-aid *and *dpb2-aid *also showed defective growth on auxin plates, whereas *hsk1-i-aid*, *ssl3-i-aid *and *cia1-i-aid *showed no defect (Table 1). We examined whether the *i*-AID system affected the amount of Mcm10-aid protein, because the above assays had indicated no apparent defect for strain *mcm10-i-aid*. The results of immunoblotting with anti-Mcm10 antibody showed that the amount of Mcm10-aid protein decreased to less than 25% of the original amount within 1 h after addition of auxin (Figure 5C), demonstrating that the *i*-AID system induced degradation of Mcm10-aid protein. Auxin-induced decrease in the tagged protein was also observed in *ssl3-i-aid *and *cia1-i-aid *strains (data not shown). Although the *i*-AID system can induce degradation of various proteins in fission yeast, further efficient depletion, which results in tight arrest of the cell cycle, will be required to analyze the functions of proteins at the molecular level.

**Table 1:** Auxin-induced growth defects in aid-tagged strains

Gene	Protein function	AID	*i-*AID	*off-*AID
*mcm4*	MCM subunit	+*	++*	NT
*orc2*	ORC subunit	NT	++*	NT
*orc4*	ORC subunit	NT	++*	++*
*orc6*	ORC subunit	NT	+*	NT
*psf1*	GINS subunit	NT	+*	++*
*pol1*	catalytic subunit of Polα	NT	-*	++*
*pol12*	second largest subunit of Polα	NT	+*	++*
*spp2*	primase subunit of Polα	NT	+*	++*
*cdc20*	catalytic subunit of Polε	NT	-*	++*
*dpb2*	second largest subunit of Polε	NT	+*	++*
*cdc45*	replication factor	NT	+*	++*
*mcm10*	replication factor	NT	-	++*
*hsk1*	catalytic subunit of DDK	NT	-	++
*ssl3*	cohesin loader	NT	-	++
*cia1*	histone chaperone	NT	-	++

++: severe growth defect on plates containing 0.5 mM auxin for AID and *i-*AID, or 0.5 mM auxin with 10 μg/ml thiamine for *off-*AID, +: slow growth on plates containing 0.5 mM auxin, -: no effect, NT: not tested, *: defect of DNA replication observed in flow cytometry analysis

### Efficient depletion of replication proteins by the *off*-AID system causes tight cell-cycle arrest

**Figure 6:**
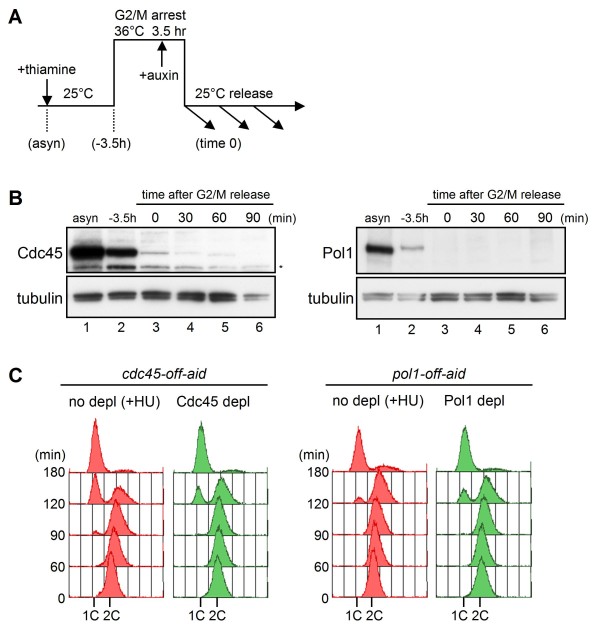
**Depletion of Cdc45 or Pol1 by the *off*-AID system causes tight cell cycle arrest in early S phase**. (A) HM3390 *cdc25-22 P*_*nmt81*_*-cdc45-aid double-TIR1 *or HM3397 *cdc25-22 P*_*nmt81*_*-pol1-aid double-TIR1 *strain grown at 25°C was cultured in the presence of thiamine (10 μg/ml) for 4 h or 6 h, respectively, arrested at G2/M phase by incubation at 36°C for 3.5 h, and then released at 25°C (time 0). NAA (0.5 mM) was added 1 h before release. HU (12 mM) was added to non-depleted cells at time 0 (not shown in the figure). (B) Cdc45-aid and Pol1-aid proteins in whole-cell extracts prepared at the indicated time points were analyzed by immunoblotting using anti-Cdc45 and anti-IAA17 (Pol1) antibodies, respectively. Non-specific band is indicated by an asterisk (*). (C) DNA contents of the cells with (right) or without (left) protein depletion were analyzed by flow cytometry.

Although the *i*-AID system promoted rapid degradation of various target proteins, significant amounts of protein remained after depletion (Figure 5C and data not shown), probably due to *de novo *protein synthesis. In order to decrease the target protein further, we combined the *i*-AID system with transcriptional repression (*off*-AID system). To achieve this, the *pol1-aid *and *cdc45-aid *genes were placed under the control of the thiamine-repressible *P*_*nmt81 *_promoter. In addition, both AtTIR1 and *Oryza sativa *TIR1 (OsTIR1), which induces more efficient degradation than AtTIR1 in budding yeast [11], were expressed from constitutive *P*_*adh15 *_promoters (*double-TIR1*). Thiamine was added to shut off the transcription of the *pol1-aid *or *cdc45-aid *gene, and then auxin was added during cell cycle arrest at the G2/M boundary using *cdc25-22 *mutation (Figure 6A). Upon release from G2/M at 1 h after addition of auxin, the amounts of Pol1-aid and Cdc45-aid proteins were greatly decreased to nearly undetectable levels (Figure 6B). The results of flow cytometry showed that *pol1-off-aid *and *cdc45-off-aid *cells were arrested with a 1C DNA content, indicating severe defects in the early stage of DNA replication (Figure 6C). The *off*-AID system promoted strong growth defect on auxin plates for almost all the strains tested including *mcm10-off-aid*, *hsk1-off-aid*, *ssl3-off-aid*, and *cia1-off-aid*, which did not show significant defect with the *i*-AID system (Table 1). These results suggest that the *off*-AID system consisting of the *i*-AID combined with promoter shut-off of the target gene is widely applicable for efficient depletion of proteins in fission yeast cells.

## Discussion

We have developed a powerful protein depletion system, "*off*-AID", in fission yeast by combining transcription repression with auxin-dependent protein degradation. An initial attempt that involved expression of TIR1 in strain *mcm4-aid *showed only a marginal defect on cell growth in the presence of auxin. However, the *i*-AID system consisting of TIR1 fused with fission yeast Skp1 markedly increased the efficiency of Mcm4-aid protein degradation, causing severe replication defect and cell cycle arrest. This suggested that the interaction between plant TIR1 and fission yeast Skp1 might be rate-limiting for SCF^TIR1 ^assembly. We also showed that the level of TIR1 expression is crucial for efficient depletion of Mcm4-aid protein as observed in plant cells [14,25]. Expression of Skp1-TIR1-NLS from the *adh15 *promoter resulted in severely defective growth on an auxin plate, whereas a reduced cellular concentration of Skp1-TIR1-NLS expressed from the *nmt41 *promoter did not result in the same phenotype (Figure 3). We noticed that the NAA concentration is also important for the AID system in fission yeast. The *mcm4-i-aid *strain did not show growth defect at 0.1 mM NAA, whereas NAA concentration higher than 1 mM affected the growth of untagged strain (data not shown). Since auxin may act as a signaling molecule to promote morphogenesis in budding yeast [34], a similar pathway may present in fission yeast. The *i*-AID system decreased the level of Mcm4-aid protein to about 10% of the original amount at 1 h after auxin addition (Figure 3), suggesting the utility of this system for depletion of the protein within a short period during the cell cycle. Although the *i*-AID system resulted in retardation of DNA replication and cell growth for more than half (11 among 15) of essential factors tested, significant defect was not observed for the *mcm10-i-aid*, *hsk1-i-aid*, *ssl3-i-aid and cia1-i-aid *(Figure 5 and Table 1). For the latter cases, the amount of remaining tagged protein seems to be higher (20-30% of the native amount) than those showing the defect (Figure 5C and data not shown). Difference in degradation efficiency might be caused by the affinity of TIR1 with the target proteins or the efficiency of ubiquitination in different targets. In addition, whether or not depletion of a protein causes severe phenotype may depend on the amount of protein required for its function. However, for the proteins that were not efficiently depleted by the *i*-AID system, the *off*-AID system that combines transcription repression with the *i*-AID system would be advantageous. The *off*-AID system promoted a reduction of the Cdc45-aid and Pol1-aid proteins to almost undetectable levels, causing severe defects in DNA replication. In addition, essential proteins unrelated to replication such as Ssl3 that is involved in sister chromatid cohesion and Cia1/Asf1 required for histone deposition can be depleted by the *off-*AID system to cause severe growth defects (Table 1). Therefore, this approach appears to be widely applicable for *in vivo *protein depletion in fission yeast.

In comparison of the AID system with previously published strategies, the AID system has several advantages. Comparing the flow cytometry results by depletion of the same target protein Mcm4, *mcm4-i-aid *cells remained with a sharp 1C DNA peak as long as 8 hr, whereas DNA content significantly increased after 3 hr incubation in *mcm4ts-td *cells (Figure 3F and [10]), suggesting that the *i*-AID system achieved tight cell cycle arrest in early S phase. In contrast to *ts*-degron, which requires high-temperature shift or sometimes temperature-sensitive allele of the target gene, the AID system promotes the degradation by addition of a synthetic auxin to the culture. This allows use of the *cdc25-22 *temperature-sensitive mutation for cell cycle synchronization. Furthermore, the *off*-AID system causes more extensive depletion than the *i*-AID system alone (Figure 6 and data not shown). On the other hand, there are some disadvantages of the *off*-AID system. It requires two-step modifications of target genes; fusion of the aid-tag sequence at the C-terminus and replacement of the promoter with the *nmt81 *promoter. The N-terminally aid-tagged *pol1-Noff-aid*, *cdc20-Noff-aid *and *mcm10-Noff-*aid strains, which were constructed by one-step replacement of the N-terminus of the gene, did not exhibit significant defect in cell growth or DNA replication (data not shown). A limitation of the AID system we noticed is that the degradation efficiency decreases at high temperature (36°C). Although use of OsTIR1 instead of AtTIR1 have overcome this problem in budding yeast and DT40 AID systems [11], *mcm4-i-aid *strain expressing *P*_*adh15*_*-skp1-OsTIR1 *in fission yeast did not improve degradation efficiency at 36°C (data not shown).

A remarkable feature of the *i*-AID system was that it selectively degraded Mcm4-aid protein from the MCM complex without depleting the other subunits (Figure 4). This feature would make the system suitable for functional analysis of single components of a large complex. This property is attributable to the ubiquitin-mediated protein degradation system, as reported for the *ts*-degron system in budding yeast [5]. It was also notable that the *i*-AID system degraded Mcm4-aid protein not only in the soluble fraction but also in the chromatin-bound MCM complex (Figure 4). In HU-arrested cells, where the replication machinery stalls near the replication origin, depletion of Mcm4-aid protein caused dissociation of Mcm6 from chromatin, probably along with the other subunits. Consistently, replication did not resume after removal of HU, because the MCM complex is required as a replicative helicase for fork progression (Figure 4). Therefore, depletion of a component of the chromatin-bound complex, such as the replication fork, would be a powerful tool for analyzing the functions of chromatin proteins at specific stages of the reaction. It is likely that cytosolic proteins in fission yeast are also susceptible to auxin-dependent degradation, as has been described [11], although we did not test them in the present study.

The *mcm4-i-aid *strain exhibited the most marked defects in growth and replication upon addition of auxin among the aid-tagged strains tested (Table 1). This is probably because a significantly large number of MCM complexes need to be loaded onto several hundred replication origins on the chromosomes [35] within the short G1 phase in the fission yeast cell cycle. Once the MCM complexes on chromatin have dissociated through degradation of Mcm4-aid protein in S phase, cells are unable to resume DNA replication because loading of the MCM complex onto chromatin is strongly inhibited after onset of S phase in order to avoid re-replication [36].

## Conclusion

We provided an improved auxin-indusible degron system for fission yeast. The *i*-AID system, where TIR1 is fused with fission yeast Skp1, greatly enhanced degradation efficiency of Mcm4-aid protein, and the *off*-AID system, which combines the *i*-AID system with transcription repression, successfully depleted Cdc45-aid and Pol1-aid proteins causing arrest at early S phase. The *off*-AID system is a powerful method for depletion of specific proteins within fission yeast cells.

## Methods

### Strains and media

*Schizosaccharomyces pombe *strains used in this study are listed in Table 2. Constructions of strains HM1813, HM1905, HM2473, HM2475, HM2491, HM2550, HM2551, HM2552, HM2572, HM2575, HM2578, HM3150 and HM3325 are described in detail below. Media used for fission yeast cultivation were YE medium as a complete medium and EMM medium as a selection medium [37]. All solid media contained 2.0% agar. Transcription from the *nmt *promoter was induced in the absence of thiamine. Transformation of *S. pombe *was performed by the lithium acetate method [38] and hygromycin B (Wako) and ClonNAT (WERNER BioAgents) were used for selection of transformants at final concentrations of 150 μg/ml and 100 μg/ml, respectively. EMM medium was used for selection with clonNAT, while PMG medium was used for hygromycin B because of higher sensitivity to hygromycin B on PMG plates than EMM plates. A synthetic auxin, NAA (1-naphthaleneacetic acid), (Nacalai Tesque), which is commercially available at a low cost (one dollar per gram), was added at a concentration of 0.5 mM to YE or EMM medium.

**Table 2:** Fission yeast strains used in this study

Strain name	Genotype	Source
972	*h*^-^	Lab stock
TNF47	*h*^- ^*ade6X ura4-D18*	Lab stock
HM1813	*h*^- ^*ade6::ade6*^+^*-P*_*nmt41*_*-AtTIR1-9myc ura4-D18*	This work
HM1905	*h*^- ^*ade6::ade6*^+^*-P*_*nmt41*_*-AtTIR1-9myc mcm4::mcm4-2HA-IAA17*	This work
HM1909	*h*^- ^*mcm4::mcm4-2HA-IAA17*	This work
HM1910	*h*^+ ^*mcm4::mcm4-2HA-IAA17 leu1-32*	This work
HM2423	*h*^- ^*mcm4::mcm4-2HA-IAA17 ade6X*	This work
HM2468	*h*^- ^*ade6::ade6*^+^*-P*_*adh15*_*-skp1-AtTIR1-2NLS-9myc ura4-D18*	This work
HM2473	*h*^- ^*ade6::ade6*^+^*-P*_*adh15*_*-skp1-AtTIR1-2NLS-9myc mcm4::mcm4-2HA-IAA17*	This work
HM2475	*h*^- ^*ade6::ade6*^+^*-P*_*adh81*_*-skp1-AtTIR1-2NLS-9myc mcm4::mcm4-2HA-IAA17*	This work
HM2491	*h*^- ^*ade6::ade6*^+^*-P*_*nmt41*_*-skp1-AtTIR1-2NLS-9myc mcm4::mcm4-2HA-IAA17*	This work
HM2550	*h*^- ^*ade6::ade6*^+^*-P*_*adh15*_*-skp1-AtTIR1-2NLS-9myc mcm10::mcm10-IAA17*	This work
HM2551	*h*^- ^*ade6::ade6*^+^*-P*_*adh15*_*-skp1-AtTIR1-2NLS-9myc cdc20::cdc20-IAA17*	This work
HM2552	*h*^- ^*ade6::ade6*^+^*-P*_*adh15*_*-skp1-AtTIR1-2NLS-9myc pol1::pol1-IAA17*	This work
HM2572	*h*^- ^*ade6::ade6*^+^*-P*_*adh15*_*-skp1-AtTIR1-2NLS-9myc orc2::orc2-IAA17*	This work
HM2575	*h*^- ^*ade6::ade6*^+^*-P*_*adh15*_*-skp1-AtTIR1-2NLS-9myc orc6::orc6-IAA17*	This work
HM2578	*h*^- ^*ade6::ade6*^+^*-P*_*adh15*_*-skp1-AtTIR1-2NLS-9myc cdc45::cdc45-IAA17*	This work
HM2580	*h*^- ^*cdc45::cdc45-IAA17*	This work
HM2985	*h*^- ^*ade6::ade6*^+^*-P*_*adh15*_*-skp1-OsTIR1-natMX6-P*_*adh15*_*-skp1-AtTIR1-2NLS ura4-D18*	This work
HM3086	*h*^- ^*cdc25-22 ade6::ade6*^+^*-P*_*adh15*_*-skp1-AtTIR1-2NLS-9myc mcm4::mcm4-2HA-IAA17*	This work
HM3150	*h*^- ^*ade6::ade6*^+^*-P*_*adh15*_*-skp1-AtTIR1-2NLS pol1::hphMX6-P*_*nmt81*_*-pol1-IAA17*	This work
HM3325	*h*^- ^*cdc45::hphMX6-P*_*nmt81*_*-cdc45-IAA17*	This work
HM3390	*h*^- ^*cdc25-22 cdc45::hphMX6-P*_*nmt81*_*-cdc45-IAA17 mcm10::6flag-mcm10 ade6::ade6*^+^*-P*_*adh15*_*-skp1-OsTIR1-natMX6-P*_*adh15*_*-skp1-AtTIR1-2NLS*	This work
HM3397	*h*^- ^*cdc25-22 pol1::hphMX6-P*_*nmt81*_*-pol1-IAA17 mcm10::6flag-mcm10 ade6::ade6*^+^*-P*_*adh15*_*-skp1-OsTIR1-natMX6-P*_*adh15*_*-skp1-AtTIR1-2NLS*	This work
MKF53	*h*^+ ^*mcm4::mcm4-2HA-IAA17 leu1-32 pREP41D*	This work
MKF54	*h*^+ ^*mcm4::mcm4-2HA-IAA17 leu1-32 pREP41D-AtTIR1-9myc*	This work
MKF55	*h*^+ ^*mcm4::mcm4-2HA-IAA17 leu1-32 pREP41D-AtTIR1-2NLS-9myc*	This work
MKF60	*h*^+ ^*mcm4::mcm4-2HA-IAA17 leu1-32 pREP41D-skp1-AtTIR1-9myc*	This work

### Construction of P_nmt41_-AtTIR1 strain

To introduce *AtTIR1 *gene under control of P_nmt41_, *AtTIR1-9myc *sequence was introduced between the *nmt41 *promoter and terminator sequences [39] on pUC-nmt41 to generate pKM15. Then a 4.4 kb *Not*I fragment from pKM15 containing *P*_*nmt41*_*-AtTIR1-9myc-T*_*nmt1 *_from pKM15 was inserted into the *Not*I site downstream of *ade6*^+ ^in pKM17 to generate pKM21. pKM21 was digested by *Eco*RI and used for transformation of TNF47 (*h*^- ^*ade6X ura4-D18*) to gain HM1813 (*h*^- ^*P*_*nmt41*_*-AtTIR1-9myc ura4-D18*). Integration of the fragment was confirmed by PCR.

### Construction of mcm4-aid strains

To fuse the aid-tag (IAA17) at the C-terminal of *mcm4*^+^, fragments containing the C-terminal region of *mcm4*^+ ^(814- to 911-amino acid) and the 3'-UTR (7 to 340 bp downstream of the open reading frame) were amplified by PCR using the primers mcm4C-F and mcm4C-R, and mcm4dw-F and mcm4dw-R2 (Table 3), respectively. The C-terminal fragment was cloned into the *Nde*I-*Sma*I site of pUC-nmt1, resulting in pKM37. The 3'-UTR fragment was introduced into the *Kpn*I-*Bam*HI site of the plasmid containing the *ura4*^+ ^gene to generate pKM38. The N-terminally 2HA-tagged IAA17 fragment (*2HA-aid*) was amplified by PCR using the primers IAA17-2HA-F and IAA17-2HA-R (Table 3) and pRS304 as a template, and then introduced at the C-terminus of *mcm4 *on pKM37 to generate pKM39. The *mcm4C-2HA-aid *fragment from pKM39 was cloned into pKM38 to create pKM40, containing *mcm4C-2HA-aid-ura4*^+^*-mcm4DW*. *Sal*I-*Xho*I-digested pKM40 was used for transformation of HM1813 (*h*^- ^*P*_*nmt41*_*-AtTIR1-9myc ura4-D18*), resulting in HM1905 (*h*^- ^*P*_*nmt41*_*-AtTIR1-9myc mcm4-2HA-aid*). Integration of *mcm4-2HA-aid-ura4*^+ ^at the *mcm4*^+ ^locus was confirmed by PCR.

**Table 3:** Primers used in this study

Primer name	Sequence
IAA17-2HA-R	5'-AAAATGCATTGCTGCAGCTCGAGCTCTGCTCTTGCAC-3'
IAA17-2HA-F	5'-AAAATGCATGCTGCAGCTCGAGCATACCCATACGATGTACCTGATTATGC TGGTTATCCTTATGATGTTCCAGACTATGCTATGATGGGCAGTGTCGAG-3'
mcm4C-F	5'-GGAATTCCATATGACTAGTGCAACTGATCCTGCAACAGGA-3'
mcm4C-R	5'-TTCCCCCGGGTTATCGTCGACTACCTGCAGGATCAGTATGTGCAATTGAA CGTAC-3'
mcm4dw-F	5'-GGGGTACCGAATTCAGCGAATAAGCTGGTATTATTCATGAGC-3'
mcm4dw-R2	5'-GGAAGATCTGGATCCTCGAGCTACGAATCATGGCGAATTATTGGTTACG-3'
skp1-F	5'-CCATCGATCATATGGCTAGCCCTGCAGGGATGTCCAAAATCAAACTGATT TCATCTGAC-3'
skp1-R	5'-GGAATTCACTAGTCAGATCTCCTGCGCCGGCTCCAGCACCAAGATCTGG GATCCCTCTGTCTTCGGCCC-3'
cdc20-IAA	5'-TCGACACTGCCCATCATAGCTGCGGAAGTTGAGTTCAGCACAGAAAGTA-3'
cdc20-ura	5'-GTTTCGTCAATATCACAAGCCTCGCCTGACCATGAGC-3'
cdc20-dw-R	5'-CGAACGTTTAAGAGCATG-3'
cdc20C-F	5'-GACATGGGGACCTTGGTG-3'
mcm10-IAA	5'-TCGACACTGCCCATCATAGCAGCGGAAGTTGAGGGAACTATTTCTAAGTC-3'
mcm10-ura	5'-GTTTCGTCAATATCACAAGCGACTTAGAAATAGTTCCC-3'
mcm10-dw-R	5'-GCTTACAAGCCCATCATACC-3'
mcm10C-F	5'-CCCTAAATCCTCTCTACC-3'
cdc45-IAA	5'-TCGACACTGCCCATCATCGTTCCTTGTGGTAGTGTTTTGAAGGACAGAC-3'
cdc45-ura	5'-GTTTCGTCAATATCACAAGCTCCACACCAGCAATTGTT-3'
cdc45-dw-R	5'-TGACTGATCCAGAATCGG-3'
cdc45C-F	5'-GCATCCCCTTGCGTTAAC-3'
orc2-IAA	5'-TCGACACTGCCCATCATCGTTCCTTGTGGTACGTCTTCCATCATATCC-3'
orc2-ura	5'-GTTTCGTCAATATCACAAGCGGACCCTTTTAGACTAGG-3'
orc2-dw-R	5'-TACAATGCTGATACTAGGA-3'
orc2C-F	5'-TCTCTATTCTTTGCCCGCC-3'
orc6-IAA	5'-TCGACACTGCCCATCATCGTTCCTTGTGGTGAAGCAGTACCATCTTTTTC-3'
orc6-ura	5'-GTTTCGTCAATATCACAAGCCGGGTACACGATATCTTTAG-3'
orc6-dw-R	5'-CAATGATTCTCAAGAGACG-3'
orc6C-F	5'-ACTAGTATCGGCAAAAGCTTTTG-3'
pol1-IAA	5'-TCGACACTGCCCATCATCGTTCCTTGTGGCGATGAAAATATCAGTCCC-3'
pol1-ura	5'-GTTTCGTCAATATCACAAGCCTACCCGTTTAAGTAATCTAC-3'
pol1-dw-R	5'-GTCAAACATGTAGTGAGTAC-3'
pol1C-F	5'-CTCATCTCAACTCAGAGA-3'
TIR1C-BmSc	5'-CGGGATCCGCGGAGACAGTGACTTAGGCAT-3'
TIR1-NLS-R	5'-CGGGATCCTTAGTCGACCACTTTGCGTTTTTTCTTTGG-3'
pol1N-nmt-F	5'-CTTATAGTCGCTTTGTTAAATCATATGAGAAAGAGAAACGCGGG-3'
pol1up-F-Spe	5'-GGACTAGTCGTGCTTCAAGTATTTCCCG-3'
pol1up-R-Bgl	5'-GAAGATCTGTTCACGAGAAGACTTTAAAG-3'
pol1dw-R-Bm	5'-CGGGATCCCGACCAGACGATCCTTTTTC-3'
cdc45up-F-Spe	5'-GGACTAGTCGTTGTGCACATGTTCACCT-3'
cdc45up-R-Bm	5'-CGGGATCCACGTTACTGGTGGTGGATCA-3'
ars2004 region-273F	5'-CGGATCCGTAATCCCACAAA-3'
ars2004 region-338R	5'-TTTGCTTACATTTTCGGGAACTTA-3'
nonARS1 region-514F	5'-TACGCGACGAACCTTGCATAT-3'
nonARS1 region-583R	5'-TTATCAGACCATGGAGCCCAT-3'

### Construction of plasmids carrying modified TIR1

The *AtTIR1-9myc *and *AtTIR1-NLS-9myc *genes were inserted between the *nmt41 *promoter and terminator sequences [39] on pREP41-Dual to generate pKM7 and pKM45, respectively.

To fuse fission yeast Skp1 at the N-terminus of AtTIR1, the *skp1 *gene lacking introns was PCR-amplified from a cDNA library (donated by H. Nojima) with a 16-amino-acid linker (Gly-Ile-Pro-Asp-Leu-Gly-Ala-Gly-Ala-Gly-Ala-Gly-Asp-Leu-Thr-Ser) at the C-terminal of the protein using the primers skp1-F and skp1-R (Table 3), then cloned into the *Cla*I-*Eco*RI site of pBluescriptII SK(+), resulting in pKM44. The *skp1 *fragment from pKM44 together with the *AtTIR1-9myc *fragment was inserted between the *nmt41 *promoter and terminator sequence on pREP41-Dual to create pKM71.

pREP41-Dual, pKM7, pKM45, and pKM71 plasmids were introduced into HM1910 (*h*^+ ^*mcm4-2HA-aid leu1-32*) by electroporation to gain MKF53, MKF54, MKF55 and MKF60, respectively [40].

### Construction of TIR1 integrant strains

For integration of the *skp1-AtTIR1-NLS-9myc *gene under control of the *nmt41 *promoter into the *ade6*^+ ^locus, the *skp1 *fragment together with an *AtTIR1-NLS-9myc *fragment was inserted between the *nmt41 *promoter and terminator sequences on pUC-nmt41, resulting in pKM82. Then *Not*I fragment containing *P*_*nmt41*_*-skp1-AtTIR1-NLS-9myc-T*_*nmt1 *_from pKM82 was inserted into the *Not*I site downstream of *ade6*^+ ^in pKM17 to generate pKM84.

For construction of the *skp1-AtTIR1-NLS *gene under control of weak derivatives of the *adh1 *promoter, the *nmt41 *promoter of pKM84 was replaced by promoter fragments from pRAD15 and pRAD81 (provided by Y. Watanabe), resulting in pKM104 and pKM105, respectively. Then pKM84, pKM104 and pKM105 were digested by *Eco*RI and used for transformation of HM2423 (*h*^- ^*mcm4-2HA-aid ade6X*) to generate HM2491 (*h*^- ^*mcm4-2HA-aid ade6*^+^*-P*_*nmt41*_-*skp1-AtTIR1-NLS-9myc*), HM2473 (*h*^- ^*mcm4-2HA-aid ade6*^+^*-P*_*adh15*_-*skp1-AtTIR1-NLS-9myc*) and HM2475 (*h*^- ^*mcm4-2HA-aid ade6*^+^*-P*_*adh81*_-*skp1-AtTIR1-NLS-9myc*), respectively. Integration of *skp1-AtTIR1-NLS-9myc *at the *ade6*^+ ^locus was confirmed by genomic PCR.

### Construction of aid-tagged derivatives of replication factors

The *mcm10*^+ ^gene was C-terminally tagged with IAA17 peptide using PCR. The integration cassette was amplified by two-step PCR amplification. The first PCR amplified fragments from the 972 (*h*^-^, wild type) genome containing the C-terminal region of *mcm10*^+ ^linked with part of the aid-tag, and the 3'-UTR of *mcm10*^+ ^linked with part of the selection marker (*ura4*^+^) gene, using the primer sets mcm10C-F and mcm10-IAA, and mcm10-ura and mcm10-dw-R (Table 3), respectively. The second PCR amplified the integration cassette from pKM40 with the primers mcm10C-F and mcm10-dw-R, and the two short fragments made by the first PCR reactions. The products of the second PCR were then used for transformation of HM2468 (*h*^- ^*P*_*adh15*_*-skp1-AtTIR1-NLS-9myc ura4-D18*). Transformants were selected on an EMM plate, and the integration of the aid-tag was confirmed by genomic sequencing. The resulting strain, HM2550 (*h*^- ^*P*_*adh15*_*-skp1-AtTIR1-NLS-9myc mcm10-aid*), showed wild-type growth, suggesting that the aid-tagged Mcm10 was functional.

HM2551 (*h*^- ^*P*_*adh15*_*-skp1-AtTIR1-NLS-9myc cdc20-aid*), HM2552 (*h*^- ^*P*_*adh15*_*-skp1-AtTIR1-NLS-9myc pol1-aid*), HM2572 (*h*^- ^*P*_*adh15*_*-skp1-AtTIR1-NLS-9myc orc2-aid*), HM2575 (*h*^- ^*P*_*adh15*_*-skp1-AtTIR1-NLS-9myc orc6-aid*), and HM2578 (*h*^- ^*P*_*adh15*_*-skp1-AtTIR1-NLS-9myc cdc45-aid*) were constructed similarly using pKM40 and the following primers (Table 3). HM2551: cdc20C-F and cdc20-IAA; cdc20-ura and cdc20-dw-R, HM2552: pol1C-F and pol1-IAA; pol1-ura and pol1-dw-R, HM2572: orc2C-F and orc2-IAA; orc2-ura and orc2-dw-R, HM2575: orc6C-F and orc6-IAA; orc6-ura and orc6-dw-R, and HM2578: cdc45C-F and cdc45-IAA; cdc45-ura and cdc45-dw-R. All the strains showed wild-type growth, except for HM2578, which grew slightly more slowly in the absence of auxin.

### Construction of the double-TIR1 strain

For integration of the *skp1-OsTIR1-NLS-9myc *gene under control of the *adh15 *promoter into the *ade6*^+ ^locus, a 1.7-kb *Xba*I-*Sal*I fragment containing the *OsTIR1 *gene from pNHK33 and a 0.4 kb *Sal*I-*Sma*I fragment containing *9myc *were introduced into the *Spe*I-*Sma*I sites of pKM104 to generate pKM111. pKM111 was digested by *Eco*RI and used for transformation.

To remove the 9myc-tag at the C-terminus of AtTIR1, the C-terminal region of AtTIR1-NLS without myc-tag was PCR-amplified using the primers TIR1C-BmSc and TIR1-NLS-R (Table 3), and the products obtained by *Bam*HI digestion were used to generate pKM132, which contains the C-terminal region of *AtTIR1-NLS *and the transcription terminator *T*_*nmt*_. The C-terminal region of *skp1-AtTIR1-NLS-9myc *was replaced by the *Nsi*I-*Sma*I fragment from pKM132, to generate pKM136 carrying *P*_*adh15*_*-skp1-AtTIR1-NLS-T*_*nmt1*_.

To construct the *double-TIR1 *strain, the *Spe*I-*Not*I fragment from pKM132 containing the C-terminal region of *AtTIR1-NLS *and *T*_*nmt1 *_was inserted into pKM126 containing the 3'-UTR of *ade6*^+^, resulting in pKM135. Then the *Xho*I-*Sal*I fragment containing the *OsTIR1 *gene was introduced into the *Sal*I site of pKM135 to create pKM143, carrying *OsTIR1-T*_*nmt1 *_and the 3'-UTR of *ade6*^+^. Then the selection marker gene, *natMX6*, cloned from pFA6a-natMX6 and the *P*_*adh15*_*-skp1-AtTIR1-NLS-T*_*nmt1 *_gene from pKM136 were inserted into the *Not*I site of pKM143 to generate pKM151. An 8.2 kb *Sac*II fragment from pKM151 containing *OsTIR1-T*_*nmt1*_, *natMX6*, *P*_*adh15*_*-skp1-AtTIR1-NLS-T*_*nmt1 *_and the 3'-UTR of *ade6*^+ ^was introduced into cells harboring *P*_*adh15*_*-skp1-OsTIR-9myc *at the *ade6*^+ ^locus to construct the *double-TIR1 *strain. A Nat^R ^transformant that grew on an EMM plate containing clonNAT (100 μg/ml) was obtained, and integration of the fragment was confirmed by genomic PCR and southern hybridization.

### Construction of P_nmt81_-pol1-aid and P_nmt81_- cdc45-aid strains

For construction of the *P*_*nmt81*_*-pol1-aid *strain, the 5'-UTR of *pol1*^+ ^was amplified by PCR using the primers pol1up-F-Spe and pol1up-R-Bgl (Table 3), and the *Spe*I-*Bgl*II-digested product was cloned into the *Spe*I-*Bam*HI sites upstream of *hphMX6 *to generate pKM154. The N-terminal region of *pol1*^+ ^was PCR-amplified using the primers pol1N-nmt-F and pol1dw-R-Bm (Table 3), and the product was digested by *Nde*I and *Bam*HI, and then cloned into the *Nde*I-*Bam*HI sites downstream of *P*_*nmt81 *_to create pKM158. Then *Sac*I fragment of pKM154 containing the 3'-UTR of *pol1*^+ ^and *hphMX6 *was introduced into the *Sac*I site of pKM158 to form pKM159. pKM159 was digested by *Eco*RV and used for transformation of HM2552 (*h*^- ^*P*_*adh15*_*-skp1-AtTIR1-NLS-9myc pol1-aid*) to create HM3150 (*h*^- ^*P*_*adh15*_*-skp1-AtTIR1-NLS-9myc P*_*nmt81*_*-pol1-aid*). The insertion was confirmed by genomic PCR.

A strain expressing Cdc45-aid from *P*_*nmt81 *_was constructed as described below. The N-terminal fragment of *cdc45*^+ ^was cloned from pGAD-Cdc45 into the *Nde*I-*Eco*RV sites of pKM158 to create pKM163. The 5'-UTR of *cdc45*^+ ^was PCR-amplified using the primers cdc45up-F-Spe and cdc45up-R-Bm (Table 3), and the product digested by *Spe*I and *Bam*HI was cloned into the *Spe*I-*Bam*HI sites upstream of *hphMX6*, resulting in pKM164. The 2.0-kb *Sac*I fragment containing the 3'-UTR of *cdc45*^+ ^and *hphMX6 *was inserted into the *Sac*I site of pKM163 to create pKM165. pKM165 was used for transformation of HM2580 (*h*^- ^*cdc45-aid*) to generate HM3325 (*h*^- ^*P*_*nmt81*_*-cdc45-aid*), and the integration was confirmed by genomic PCR.

### Preparation of cell extracts and immunoblotting

*S. pombe *cells (1 × 10^8 ^cells) were fixed with 20% TCA and suspended in 0.1 ml of urea solution (50 mM NaPi [pH 8.0], 8 M urea, 1 mM DTT, 0.1% Nonidet P-40). Cells were disrupted with acid-washed glass beads using a Micro Smash (TOMY) three times for 45 sec each time. Proteins in the extracts were separated by SDS-PAGE and transferred onto PVDF membrane (Immobilon, Millipore Corp). The membranes were incubated for 1 h at room temperature in PBST (10 mM Na_2_HPO_4_, 137 mM NaCl, 2.7 mM KCl, 1.76 mM KH_2_PO_4_, 0.1% Tween 20) containing 5% skim milk and reacted in PBST containing 1% skim milk overnight at 4°C with rabbit anti-Mcm4 antibody [41], rabbit anti-Mcm2 antibody [33], rabbit anti-Mcm3 antibody [42], rabbit anti-Mcm5 antibody [33], rabbit anti-Mcm6 antibody [33], rabbit anti-Mcm7 antibody [42], rabbit anti-Mcm10 antibody (will be published elsewhere), rabbit anti-Cdc45 antibody [43], rabbit anti-IAA17 antibody [11], mouse anti-Myc antibody 9E11 (NeoMarkers) or mouse anti-TAT1 antibody [44] at dilutions of 1:2,000, 1:1,000, 1:3,000, 1:1,000, 1:3,000, 1:1,000, 1:2,000, 1:2,000, 1:2,000, 1:1,000 and 1:500, respectively. HRP-conjugated anti-mouse or anti-rabbit immunogloblin G was used as the secondary antibody (1:10,000; Jackson). Binding was visualized with West Pico Chemiluminescent Substrate and Femto Maximum Sensitivity Substrate (Thermo).

### Cell cycle synchronization

To synchronize the cell cycle, the thermosensitive mutation *cdc25-22 *was used for G2/M arrest [26]. Derivatives carrying *cdc25-22 *were incubated at 36°C for 3 h for arrest at the G2/M boundary and then released at 25°C. To repress transcription, thiamine was added at a final concentration of 10 μg/ml at the indicated time points before G2/M arrest (see Figure legends). Synthetic auxin, NAA (1-naphthaleneacetic acid) (Nacalai Tesque), was added at a concentration of 0.5 mM, 1 h before the release from G2/M to induce protein degradation.

To arrest cells in early S phase, cells released from the G2/M boundary were cultured for 3 h in the presence of 12 mM hydroxyurea (HU, Sigma), which depletes the cellular dNTP pools. NAA was added 1 h before the release from HU arrest.

### Chromatin immunoprecipitation assay

ChIP assays were performed as described previously [45] with some modifications. Derivatives of *cdc25-22 *grown in EMM medium at 25°C for 15 h to 0.6 × 10^7 ^cells/ml were arrested at the G2/M boundary by incubation at 36°C for 3 h and then released at 25°C in the presence of 12 mM HU to retard replication forks. The cells (3 × 10^8^) were fixed in 1% formaldehyde (Sigma) for 15 min and then in 125 mM glycine for 5 min at room temperature with gentle shaking. After being washed once with cold water, the cells were suspended in 450 μl of breaking buffer (50 mM Hepes-KOH [pH 7.4], 1 mM EDTA, 140 mM NaCl, 0.1% sodium deoxycholate, 0.1% Triton X-100, 1 mM phenylmethylsulfonyl fluoride, 0.1% proteinase inhibitor cocktail [Sigma]) and disrupted with glass beads using a Micro Smash (TOMY) five times for 30 s each time. The cell extracts were recovered by centrifugation at 3,000 rpm for 10 s. After addition of 50 μl of 10% Triton X-100, the samples were sonicated four times for 10 s each time. The supernatant obtained by centrifugation at 15,000 rpm for 10 min was used for immunoprecipitation with magnet beads (Dynal) conjugated with rabbit anti-Mcm6 antibody (1:400). After incubation of the cell extracts with the beads at 4°C for 2 h, the immunoprecipitates were rinsed with ChIP lysis buffer (50 mM Hepes-KOH [pH 7.4], 1 mM EDTA, 140 mM NaCl, 0.1% sodium deoxycholate, 1% Triton X-100, 1 mM phenylmethylsulfonyl fluoride) once, ChIP lysis buffer containing 640 mM NaCl twice, ChIP wash buffer (10 mM Tris-HCl [pH 8.0], 1 mM EDTA, 250 mM LiCl, 0.5% sodium deoxycholate, 0.5% Nonidet P-40) twice and TE1 (10 mM Tris-HCl [pH 8.0], 1 mM EDTA) once, and then the obtained protein-DNA complex was eluted with TES (10 mM Tris-HCl [pH 8.0], 1 mM EDTA, 1% SDS) by incubation at 65°C for 30 min. To reverse the cross-linking, the eluates were incubated at 65°C for 15 h. The remaining proteins were digested with Proteinase K (Merck) and the DNA was purified by extraction with phenol and chloroform. The DNA recovered by ethanol precipitation was suspended in TE. DNA prepared from whole-cell extracts or immunoprecipitated fractions was analyzed by real-time PCR using SYBR green I in a 7300 real-time PCR System (Applied Biosystems). The primer sets used for real-time PCR were ars2004 region-273F and ars2004 region-338R for *ars2004*, and nonARS1 region-514F and nonARS1 region-583R for the non-origin region (Table 3).

### Flow cytometry

Cells were fixed with 70% ethanol and incubated with 0.5 μg/ml Propidium iodide and 50 μg/ml RNaseA in 50 mM sodium citrate for 1 h at 37°C. Samples were then measured using a FACScan (BECTON DICKINSON).

### Deposit of strains

Strains HM2468, HM2473 and HM2985 and plasmid pKM40 are deposited to the National BioResource Project http://yeast.lab.nig.ac.jp/nig/ and will be available upon request.

## Authors' contributions

MKan carried out the molecular genetic studies, participated in the design of the study and drafted the manuscript. KN participated in the molecular genetic studies. MKane participated in the design of the study. TK conceived of conception of the study. TT and TN participated in the design of the study and contributed to analysis and interpretation of data. HM conceived of the study, and participated in its design and coordination. All authors read and approved the final manuscript.
